# Hyperphagia and impulsivity: use of self-administered Dykens’ and in-house impulsivity questionnaires to characterize eating behaviors in children with severe and early-onset obesity

**DOI:** 10.1186/s13023-024-03085-1

**Published:** 2024-02-23

**Authors:** Lara Arnouk, Hélène Chantereau, Sophie Courbage, Patrick Tounian, Karine Clément, Christine Poitou, Béatrice Dubern

**Affiliations:** 1 Pediatric Nutrition and Gastroenterology Department, French Reference Center for Prader-Willi Syndrome and Other Rare Obesities (PRADORT), Assistance Publique Hôpitaux de Paris, Trousseau Hospital, 26 Avenue du Dr Netter, 75012 Paris, France; 2 Nutrition Department, French Reference Center for Prader-Willi Syndrome and Other Rare Obesities (PRADORT), Assistance Publique Hôpitaux de ParisPitié-Salpêtrière Hospital, Paris, France; 3 INSERM, Nutrition and Obesity: Systemic Approaches, NutriOmics, Research Unit, Sorbonne Université, Paris, France

**Keywords:** Childhood obesity, Eating behaviors, Hyperphagia, Hypothalamus, Genetics, Dykens’ questionnaire, Food impulsivity questionnaire

## Abstract

**Background:**

The determinants of early-onset obesity (< 6 years) are not completely elucidated, however eating behavior has a central role. To date no study has explored eating behavior in children with severe, early-onset obesity. Self-administered questionnaire data from these children were examined to evaluate eating behavior and the etiology of early-onset obesity.

**Methods:**

Children with severe, early-onset obesity (body mass index [BMI] > International Obesity Task Force [IOTF] 30) of different etiologies (hypothalamic obesity [HO], intellectual disability with obesity [IDO], common polygenic obesity [CO]) were prospectively included. BMI history and responses from the Dykens’ Hyperphagia Questionnaire and an in-house Impulsivity Questionnaire at first visit were compared between groups.

**Results:**

This cohort of 75 children (39 girls; mean age ± standard deviation [SD] 10.8 ± 4.4 years) had severe, early-onset obesity at an age of 3.8 ± 2.7 years, with a BMI Z-score of 4.9 ± 1.5. BMI history varied between the 3 groups, with earlier severe obesity in the HO group versus 2 other groups (BMI > IOTF40 at 3.4 ± 1.6 vs. 4.6 ± 1.6 and 8.4 ± 4.1 years for the IDO and CO groups, respectively [*P* < 0.01]). Absence of adiposity rebound was more prevalent in the HO group (87% vs. 63% and 33% for the IDO and CO groups, respectively [*P* < 0.01]). The Dykens’ mean total score for the cohort was 22.1 ± 7.2 with no significant between-group differences. Hyperphagia (Dykens’ score > 19) and impulsivity (score > 7) were found in 50 (67%) and 11 children (15%), respectively, with no difference between the HO, IDO and CO groups regarding the number of patients with hyperphagia (10 [67%], 14 [74%], and 26 [63%] children, respectively) or impulsivity (2 [13%], 1 [7%], and 8 [19%] children, respectively). Children with food impulsivity had significantly higher total and severity scores on the Dykens’ Questionnaire versus those without impulsivity.

**Conclusion:**

The Dykens’ and Impulsivity questionnaires can help diagnose severe hyperphagia with/without food impulsivity in children with early-onset obesity, regardless of disease origin. Their systematic use can allow more targeted management of food access control in clinical practice and monitor the evolution of eating behavior in the case of innovative therapeutic targeting hyperphagia.

## Background

Early-onset obesity is a complex pathology linked to a marked central impairment of weight regulation, either genetic or lesional in origin [1]. The hypothalamus plays a critical role due to its close link with the other centers regulating eating behavior and metabolism (e.g., reward systems, cortical regions, and peripheral organs). The melanocortin pathway in the hypothalamus regulates appetite and eating behavior and variations in the genes encoding the melanocortin-4 receptor (MC4R) have been linked to severe obesity in children [2]. Impairment of the hypothalamus function due to any genetic or lesional cause is responsible for global neuroendocrine pathology, named hypothalamic obesity (HO) [1, 3–6]. HO is rare (< 5% all obesities), and models exist to understand the mechanisms of early-onset HO [1, 7]. Early-onset HO should be distinguished from early-onset obesity with an intellectual disability (IDO), where the intellectual disability is not necessarily related to the cause of the obesity. Indeed, various reasons can be responsible for obesity development in case of IDO (increased food intake of high energy density foods and choice of highly calorific "pleasant" foods, increased sedentary lifestyle, reduced physical activity, psychotropic treatments). Finally, common polygenic obesity (CO) may mimic the phenotype of HO, especially in case of a high polygenic score as recently described [8]. A deeper phenotyping of BMI trajectory, associated signs, and eating behavior in early-onset obesities could allow IDO and CO to be distinguished from HO. Moreover, a better knowledge of the eating behavior of children with early-onset obesities is essential to identify children with specific clinical conditions such as HO and, thereby, propose specific care management.

Although severe hyperphagia with impulsivity and lack of control is classically described during the early years of life in specific cases of children with HO [9–15], these data are mainly descriptive, often incomplete, and have been derived from studies with variable methodologies (e.g., self-administered questionnaires, ad libitum meals, dietary interviews). Self-administered questionnaires are still commonly used for children, usually completed by the parents and/or caregivers on the children’s behalf when needed. Dykens’ Hyperphagia Questionnaire, for example, was originally developed for patients with Prader-Willi Syndrome (PWS) and explores dimensions such as active foraging and food obsession [10]. It has also been used in other rare genetic obesities to evaluate the severity of hyperphagia [12, 13, 16]. Other questionnaires such as the Child Eating Behavior Questionnaire may also be used depending on the study and age group [14, 17]. In the absence of a questionnaire specifically dedicated to early-onset obesity, the development of local assessment tools is sometimes necessary. Irrespective of the method used, the recent development of new therapies targeting hyperphagia, such as MC4R agonists, now makes simple assessment of eating behavior essential in current practice [18, 19].

The aim of our study was to explore the eating behavior of 3 groups of children with early-onset and severe obesity of different origins (HO compared with IDO and CO) using 2 different self-administered questionnaires to examine if there are any unique features that can help to distinguish the different obesity etiologies. For this purpose, we chose to use the well-known Dykens’ Hyperphagia Questionnaire and an in-house questionnaire developed by our team aiming to compare the results obtained with each of them and to explore if they could be complementary for the characterization of feeding behavior in these populations.

## Methods

We performed a prospective monocentric study in the Department of Pediatric Nutrition and Gastroenterology at Trousseau Hospital (French Reference Center PRADORT [Prader-Willi syndrome and obesity with eating disorders] Reference Centre, Paris, France). The questionnaires were completed by parents, and children when possible, during their first visit to the clinical department and after signing a consent form to participate in the French Rare Obesity Cohorts With Food Behavioral Disorders: Better Diagnosis for Better Treatment (ObeRare) cohort (NCT04604626).

Children with early-onset obesity were divided into 3 groups according to possible etiologies [1, 6]. The HO group included 15 children with an established diagnosis of genetic (syndromic or not) or lesional origin of HO (PWS [n = 2], X-fragile [n = 2], Bardet-Biedl syndrome [n = 2], 16p11.2 deletion [n = 1], *Magel-2* variant [n = 1], pseudoparathyroidism [n = 1], 22q11 deletion [n = 1], homozygous *proopiomelanocortin (POMC)* variant [n = 1], *MC4R* mutation [n = 1], and hypothalamic tumors [n = 3]). In the IDO group, 17 children with autism spectrum disorder, 1 child with Jacobsen syndrome, and 1 child with Down syndrome were included. Finally, 41 children were included in the CO group. These children had negative genetic explorations (direct sequencing of the 5 major genes [*LEP*, *LEPR*, *POMC*, *PCSK1*, *MC4R*] of the leptin/melanocortin pathway and array CHG in addition to whole exome in case of IDO) and had no hypothalamic lesion or tumor.

Phenotypic data included sex, age at first consultation, BMI (kg/m^2^) and BMI Z-score according to the French reference population [20], history of obesity with age at which BMI crosses IOTF > 30 and IOTF > 40 curves, and age at adiposity rebound (lowest point of BMI curve just before its re-ascension) [21]. Children with IDO were referred to our clinical department after an evaluation by our local geneticists and/or pediatricians specialized in neurodevelopment during childhood.

We chose to use the validated Dykens’ Hyperphagia Questionnaire [10], which explores different dimensions of hyperphagia, and an impulsivity questionnaire developed within our center which specializes in the management of children suffering from severe and early-onset obesity. Both are self-administered questionnaires with single-choice answers (i.e. completed by the patient/caregiver, rather than a healthcare professional or specialist). The Dykens’ Hyperphagia Questionnaire consists of 13 items completed by parents/caregivers to assess the child’s eating behavior and severity symptoms. These items are divided into 3 dimensions of behavior, drive and severity which explore the hyperphagic behavior (e.g., active search for food such as bargaining, manipulation to get food, stealing food), the child’s attitude (e.g., tantrums and food obsession), and the severity of this behavior, respectively. Responses to each item in the questionnaire are graded from 1 (no problem) to 5 (severe or frequent problem) except for Item 12, which gathers information on the age of onset of the disorder. Hyperphagia is diagnosed when the total score is > 19. The Dykens’ Hyperphagia Questionnaire has been shown to be robust [10], providing insights into the patient’s hyperphagia phenotype and its severity.

**Fig. 1:**
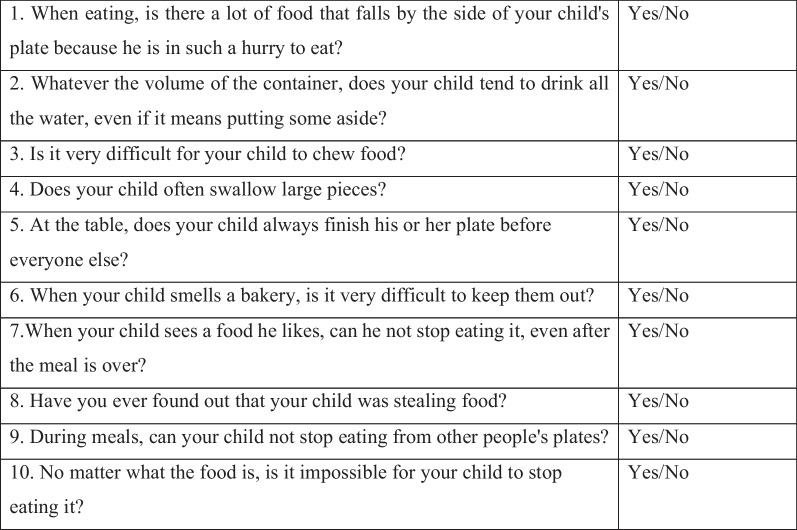
The in-house questionnaire on food impulsivity

The in-house Impulsivity Questionnaire was developed with the aim of better characterizing food impulsivity (lack of control) through 10 binary response questions (yes or no). It has been used in our clinic since 2016 in consultations before the first dietetic interview. The presence of impulsivity is identified when at least 7 or more answers are positive (Fig. 1).

The data were analyzed using Pvalue.io software. Values were expressed as mean ± SD or median (min–max) depending on the parameters. Descriptive analyses (quantitative and qualitative) used the Mann–Whitney and Welch tests. For univariate analyses, either the Kruskal–Wallis, the Pearson, or the Chi-square tests were used depending on the size of the population. Significance was denoted by *P* values < 0.05.

## Results

### Study population

We included 75 children (39 girls) from 2017 to 2020 with a mean age of 10.8 ± 4.4 years and severe obesity (mean BMI Z-score: 4.9 ± 1.5) [min–max: 4.0–5.8]. The mean age of onset of obesity (age at which BMI crossed the IOTF30 curve) was 3.8 ± 2.7 years. The BMI was above the IOTF30 curve at the age of 3 years for 34 children (45%) and at the age of 6 years for 53 children (71%). Thirty-two children (43%) had a BMI above the IOTF40 curve (severe obesity) at a mean age of 5.8 ± 3.7 years [min–max: 1.0–13.0 years]. The age at adiposity rebound was on average 2.5 ± 1.4 years [min–max: 0.2–7.0 years]. A lack of adiposity rebound, defined as a BMI curve having an ascent without slowing down before the age of 6 to 7 years, was noted in 54% of the children (data were available for 68 children only).

### BMI history according to the etiology of obesity

The comparison of the BMI history between the 3 groups showed that children with HO had more severe and early-onset obesity (Table 1) compared with the 2 other groups. The absence of adiposity rebound was also more frequent (87% of cases) in this group compared with children in the IDO (63%) or CO (33%) groups (*P* < 0.01). Similarly, the age at which BMI rose above the IOTF40 was lower in children with HO (3.4 ± 1.6 years) compared with children with IDO (4.6 ± 1.6 years) or CO (8.4 ± 4.1 years) (*P* < 0.01).

**Table 1:** Comparison of phenotype according to etiology of obesity

	HO (n = 15) mean ± SD [min–max]	ID (n = 19) mean ± SD [min–max]	CO (n = 41) mean ± SD [min–max]	*P* value
Age, years (n = 75)	**8.1 ± 4.7**	**10.9 ± 3.9**	**11.7 ± 4.1**	**0.04**
Sex ratio, M/F (n = 75)	7/8	8/11	21/20	0.80
BMI Z-score, DS (n = 75)	5.1 ± 2.6	4.6 ± 1.2	5.0 ± 1.1	0.39
Age when BMI crosses IOTF30, years (n = 64)	**2.3 ± 2.0 [0.9–3.0]**	**3.8 ± 2.0 [2.6–5.0]**	**4.3 ± 3.1 [2.0–5.7]**	**0.04**
Age when BMI crosses IOTF40, years (n = 31)	**3.4 ± 1.6 [3.0–6.0]**	**4.6 ± 1.6 [4.0–6.0]**	**8.4 ± 4.1 [6.0–13.0]**	** < 0.01**
Age at adiposity rebound, year (n = 31)	1.7 ± 1.8 [1.1–2.4]	1.9 ± 0.8 [1.5–2.0]	2.6 ± 1.4 [2.0–4.0]	0.53
Children with BMI > IOTF30 at 3 years (%) (n = 34)	10 (67)	9 (53)	14 (44)	0.34
Children with BMI > IOTF30 at 6 years (%) (n = 53)	11 (79)	16 (94)	26 (79)	0.38
Absence of adiposity rebound (%) (n = 37)	**13/15 (87)**	**12/19 (63)**	**12/36 (33)**	** < 0.01**
Dykens’, total	24.9 ± 9.2 [17–32]	23.0 ± 8.5 [20–30]	20.7 ± 5.3 [17–23]	0.11
Dykens’, hyperphagic behavior	10.6 ± 5.4	9.9 ± 4.9	8.8 ± 2.5	0.71
Dykens’, drive	10.4 ± 3.5	9.3 ± 3.1	8.9 ± 3.3	0.32
Dykens’, severity	4.2 ± 2.4	3.8 ± 2.2	3.0 ± 1.2	0.07
Dykens’, total > 19 (%)	10/15 (67)	14/19 (74)	26/41 (63)	0.63
Impulsivity > 7 (%)	2/15 (13)	1/15 (7)	8/41 (20)	ND

BMI, body mass index; CO, common obesity with early-onset; HO, hypothalamic obesity; ID, intellectual deficiency with obesity; IOTF, International Obesity Task Force; NS, not significant; SD, standard deviation; ND, not determined

Bold values indicate *P* < 0.05

### Characteristics of the eating behavior according to the etiology of obesity

In the cohort (n = 75), the mean Dykens’ total score was 22.1 ± 7.2 with a median (range) of 22.0 (6.0–39.0) which was lower than the scores initially reported in PWS (mean of 27.8 ± 4.0 in children aged 4–10 years and 30.0 ± 5.0 in 11–19 years) [10]. A total Dykens’ score > 19, considered to indicate severe hyperphagia, was noted for 50 children, i.e., 67% of cases in our cohort.

As such, the comparison of Dykens’ scores between the 3 groups showed no significant difference for the total score or any of the 3 dimensions (Table 1). The total Dykens’ score was > 19 for more than 60% of the children in the 3 groups (10/15 children with HO (67%); 14/19 children with IDO (74%), and 26/41 children with CO (63%) (not significant)).

Concerning the Impulsivity Questionnaire, a score > 7 was only noted in 11 subjects (15%). No difference between the HO, IDO and CO groups regarding the number of patients with impulsivity (2 [13%], 1 [7%], and 8 [19%] children, respectively) was noted. These children had significantly higher Dykens’ total and severity scores compared with children with a score < 7 (Table 2). Due to the small number of children, a comparison between the 3 groups could not be performed.

**Table 2:** Comparison of dimensions of Dykens’ Hyperphagia Questionnaire according to the level of impulsivity

Median [min–max]	Score > 7 (n = 11)	Score < 7 (n = 64)	*P* value
Total	**25.0 [23.5–31.5]**	**22.0 [17.8–25.0]**	**0.02**
Hyperphagic behavior	10.0 [8.0–13.0]	9.0 [6.8–11.0]	0.3
Drive	12.0 [8.5–13.5]	9.0 [6.8–11.2]	0.07
Severity	**4.0 [3.0–6.5]**	**3.0 [2.0–4.0]**	**0.04**

Bold values indicate *P* > 0.05

**Fig. 2:**
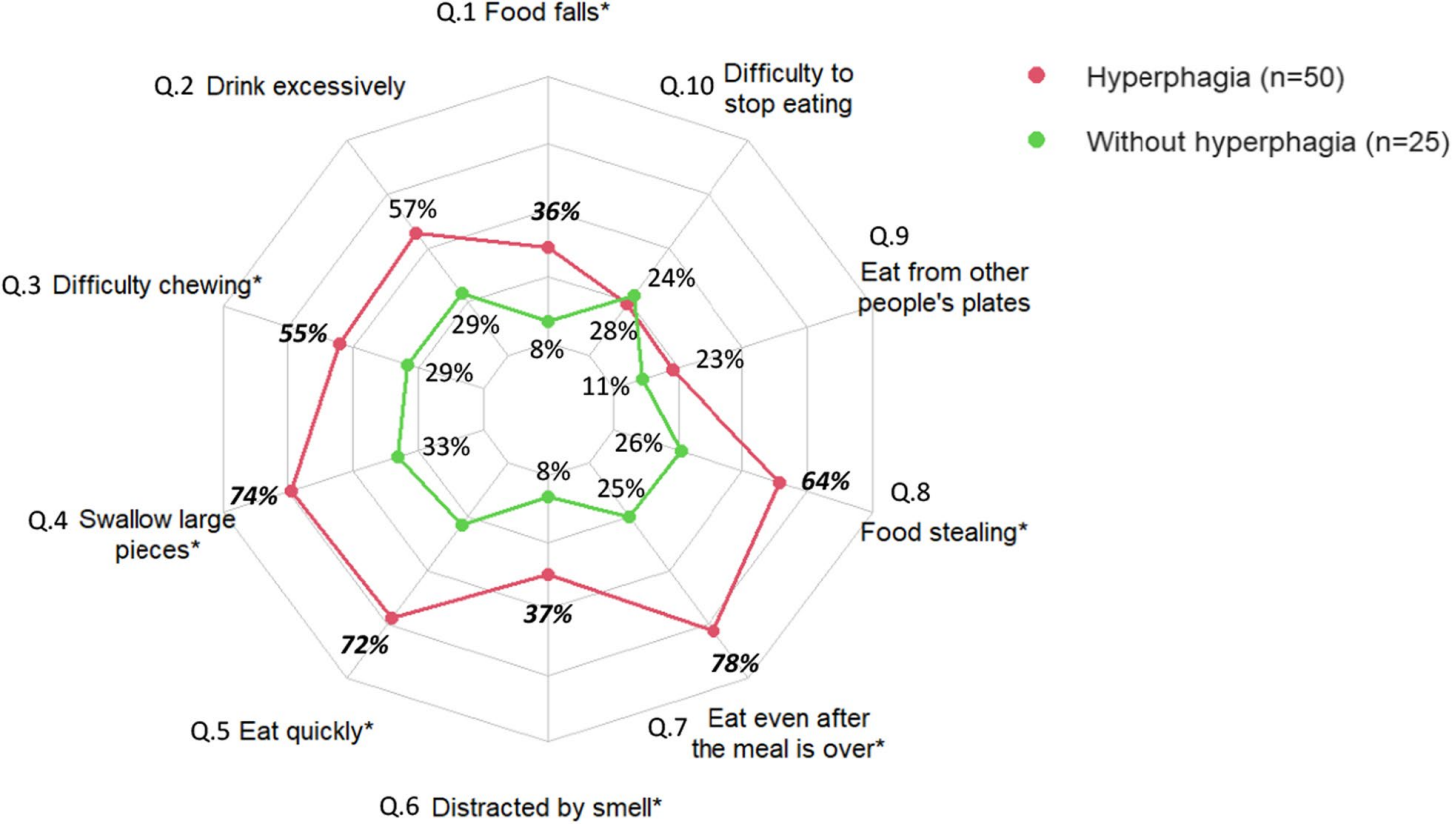
Frequency of positive responses to each question from the in-house questionnaire in case of Hyperphagia (defined as a Total Dykens’ questionnaire score of over nineteen). Q.1: “Food falls” or “When eating, is there a lot of food that falls by the side of your child’s plate because he is in such a hurry to eat?”; Q.2: “Drink excessively” or “Whatever the volume of the container, does your child tend to drink all the water, even if it means putting some aside?”; Q.3: “Difficulty in chewing” or “Is it very difficult for your child to chew food?”; Q.4: “Swallow large pieces” or “Does your child often swallow large pieces?”; Q.5: “Eat quickly” or “At the table, does your child always finish his or her plate before everyone else?”; Q.6: “Distracted by smell” or “When your child smells a bakery, is it very difficult to keep them out?”; Q.7: “Eat even after the meal is over” or “When your child sees a food he likes, can he not stop eating it, even after the meal is over?”; Q.8: “Food stealing” or “Have you ever found out that your child was stealing food?”; Q.9: “Eat from other people’s plates” or “Have you ever found out that your child was stealing food?”; Q.10: “Difficulty to stop eating” or “No matter what the food is, is it impossible for your child to stop eating it?”. Bold values indicate *P* > 0.05

Univariate analysis showed that each dimension of the Dykens’ Questionnaire was positively associated to the impulsivity score (Dykens’ total score r = 0.57; *P* < 0.001; Behavior score r = 0.47; *P* < 0.001; Drive score r = 0.50; *P* < 0.001; Severity score r = 0.3; *P* < 0.01) (n = 75). Finally, in order to search for a possible complementary relationship between the 2 questionnaires, we compared the proportion of positive responses to our in-house Impulsivity Questionnaire with the presence or absence of hyperphagia according to the Dykens’ Questionnaire. We found that a positive response to most questions of our in-house Impulsivity Questionnaire was more frequently noticed in patients with hyperphagia (Fig. 2). Interestingly, positive responses to the two questions specifically assessing the lack of control during meals and related to the general behavior (Q.9 and Q.10) (Fig. 1) were not significantly more frequent in the group with hyperphagia.

## Discussion

The children involved in this study had a severity of obesity that was identical at the time of inclusion, but children with HO had a much earlier weight gain and an absence of adiposity rebound in most cases. These results are related to the secondary alterations to the hypothalamic damage that likely induce an earlier obesity picture and continuum of a phenotype already described [1, 4, 6]. The abnormal development of high BMI with no adiposity rebound seem to be very strong elements indicative of the presence of HO and should be flagged in cases of uncontrollable hyperphagia, as described in other situations [15, 22, 23]. With a more precise analysis of the HO phenotype, an early diagnosis can be made, allowing discussion of specific treatments that target hyperphagia, such as recombinant leptin or MC4R agonists [15, 16, 24].

In this study, we explored the eating behavior of children with severe and early-onset obesity related to multiple origins (HO, IDO, or CO) using the Dykens’ Questionnaire and an in-house developed Impulsivity Questionnaire. Severe hyperphagia was found in nearly 70% of cases and was positively associated with food impulsivity, found in 15% of cases. Moreover, the questionnaire-evaluated impulsivity and the severity of hyperphagia were linked, irrespective of the etiology of obesity. Thus, children with severe obesity very early in life appear to have an eating behavior similar to that described in rare genetic syndromes such as Prader-Willi or Bardet-Biedl syndromes, sometimes but not always accompanied by food impulsivity. Intense hyperphagia that is difficult to control during childhood is a characteristic of these diseases and probably contributes to the rapid weight gain and development of severe obesity often observed.

In our study, food impulsivity was associated with more severe hyperphagia and particularly a significantly higher Dykens’ severity score in children who were developing severe early-onset obesity. As the severity dimension of the Dykens’ questionnaire explores time spent talking about food and interference with daily life [10], our results highlight the significant pre-occupation with food that children with early-onset obesity have, with difficult food control and poor tolerance of food restriction. This preoccupation is consistent with the pathophysiological mechanisms of early-onset obesity previously described [1, 4, 6]. The lack of significant differences across the impulsivity and hyperphagia scores of the HO, IDO and CO groups suggests that disordered eating behavior is a common feature in early onset obesity, even allowing for significant difference early in each child’s life. This constant pre-occupation with food largely explains the daily burden of families to control their child’s eating behavior and the major stigma expressed by adults suffering from obesity who feel ashamed of their weight, whereas the true driver of this eating behavior is the interruption of the leptin/melanocortin pathway [25, 26].

The relationship between the Dykens’ and Impulsivity Questionnaires may be complementary as each questionnaire explores different dimensions of eating behavior. The Dykens’ Questionnaire evaluates the obsession around food and any invasive thoughts around the active search for food (i.e., theft, pilfering) [10], whereas our in-house Impulsivity Questionnaire evaluates the inability to control oneself in the case of visual or olfactory stimuli, which is crucial in the management of these children even before energy restriction. This impulsivity should also be distinguished from loss-of-control eating disorders as binge eating disorders (BED), which can be observed in adults and adolescents in the context of adverse traumatic events during childhood and/or of repeated drastic energy restriction [27, 28]. In our cohort, only 15% of patients had hyperphagia associated with consistent impulsivity, which suggests that impulsivity evaluated through our in-house questionnaire could be characteristic of a more severe phenotype or to the global behavior of children suffering of early onset severe obesity.

Our work has several limitations. Firstly, it is a monocentric study with a relatively small number of patients and should be extended. The threshold of 7/10 positive answers used to define impulsivity with our Impulsivity Questionnaire remains to be validated in a larger cohort. A composite score could also be developed to explore the eating behavior of children suffering from HO; individual questions from the Dykens’, Impulsivity, or Child Eating Behavior questionnaires could be used for this purpose. Secondly, we were not able to ascertain a genetic diagnosis of some children from the IDO or the CO group: therefore, the presence of an unknown variant that alters hypothalamic functioning cannot be excluded in these children requiring additional genetic testing in other genes if they have recently described as potentially involved in these phenotypes. Thirdly, our department specializes in the management of early-onset severe obesity which partly explains the extreme obesity phenotypes observed without significant differences in eating behaviors in the HO, IDO and CO groups. Additionally, the mean age at inclusion was between 8 and 10 years of age, which probably underlies the lack of significant difference in eating behavior observed between children with HO versus children with CO or IDO. If these patients had been evaluated earlier in life during the initial stages of obesity, it can be assumed that the characteristics of eating behaviors would have been significantly different. Finally, we cannot exclude the impact of any possible underestimation of behavior or misinterpretation of the questions by patients/parents/carers, especially for the in-house Impulsivity Questionnaire.

In summary, our study showed that the Dykens’ and Impulsivity Questionnaires can be used to evaluate childhood eating behaviors and are useful to diagnose severe hyperphagia, with or without impulsivity, in children developing early obesity. This diagnosis can be made regardless of the origins of the childhood obesity. These self-administered questionnaires could be routinely used in clinical practice to allow targeted management of food-access control (e.g., avoidance of sensory stimuli to control impulsivity). We therefore suggest that these questionnaires are useful tools with which to assess and monitor childhood eating behavior and to accompany any pharmacological therapy for hyperphagia [18, 19].

## Data Availability

The datasets used and analysed during the current study are included in this published article and available from the corresponding author on reasonable request.
